# Complication of an Odontogenic Infection to an Orbital Abscess: The Role of a Medical Fraudster (“Quack”)

**Published:** 2018-05

**Authors:** Nikhil Arora, Ruchika Juneja, Ravi Meher

**Affiliations:** 1 *Department of Otorhinolaryngology and Head and Neck Surgery, Maulana Azad Medical College and Associated Lok Nayak Hospital, New Delhi-110002, India.*

**Keywords:** Facial pain, Orbital cellulitis, Tooth extraction

## Abstract

**Introduction:**

Complication of an odontogenic infection to an orbital abscess is not a common presentation. The progression from a simple toothache to a condition that may lead to loss of vision is sudden and severe.

**Case Report:**

We report a rare case in which a patient developed facial cellulitis that progressed to orbital abscess after unsterile dental manipulation by a medical fraudster (“quack”). The patient was initiated on high-grade antibiotics, which resolved the facial cellulitis. However, the patient developed orbital abscess with restricted mobility of the right eye in the lateral gaze. After radiological confirmation of the abscess, it was drained by an external approach. Due to timely intervention, the extra-ocular mobility was regained, and the vision remained unaffected.

**Conclusion:**

Knowledge of the routes of the spread of dental infection to the vital structures and the urgent need for aggressive multidisciplinary management is paramount. Furthermore, awareness of the rising quack culture in developing nations needs to be increased.

## Introduction

Orbital abscess secondary to an odontogenic cause is a very rare presentation, however, it is a cause for concern due to the risk of permanent vision loss. Early aggressive management is required for this complication. It is important to address the dental pathology early in the course of disease with aseptic precautions in order to prevent this hazardous complication. The presentation and management of an orbital abscess and the role of a medical fraudster (“quack”) in increasing the risk of such an infection is discussed in our case report.

## Case Report

**Fig 1:**
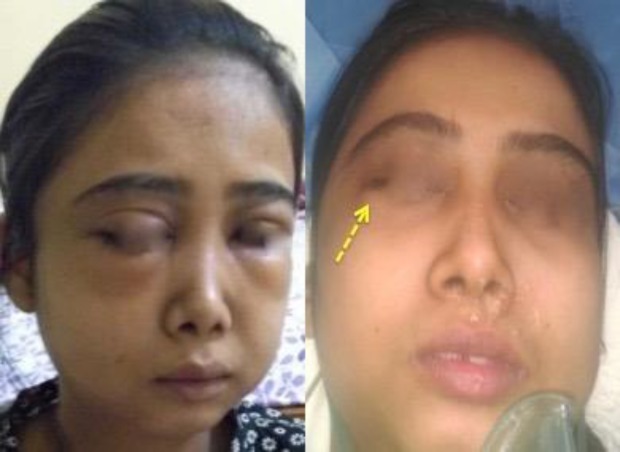
Preoperative picture of the patient with facial cellulitis and orbital swelling

A 22-year-old female presented to the Otorhinolaryngology Outpatient Department with a complaint of swelling below the right eye for the last 10 days. The patient gave a history of a tooth extraction 3 weeks previously, 2 days after which she developed severe pain and swelling over face and eyes (Fig.1). The patient was being managed conservatively at another hospital for this condition. Although there was a decrease in size of the swelling, however, it persisted even after a course of antibiotics  (Fig.1). 

On enquiring further, the patient revealed that she had had an episode of toothache 4 weeks previously, following which she sought opinion from a local quack. The quack extracted a tooth from the upper alveolus on the right side according to the patient. The facial swelling followed this surgical procedure. Examination of the eye revealed a localized swelling infero-medial to the lateral canthus and below the lower eyelid, measuring about 3×3 cm. The swelling was soft and cystic but non-fluctuant. Surprisingly, there were no signs of inflammation. Although the vision was normal, the right eye mobility was restricted along the lateral gaze. The anterior rhinoscopic examination was unremarkable without any signs of sinusitis. Oral cavity examination revealed the absence of right maxillary first premolar tooth.

**Fig 2:**
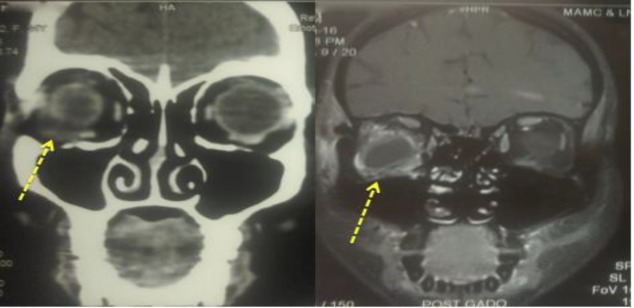
Coronal cuts of CECT and MRI with cystic swelling; likely an abscess in the right orbit

The patient retained a contrast enhanced computed tomography (CT) of the nose, paranasal sinuses and orbit from the previous hospital, which revealed a soft tissue mass lesion in the right orbit compressing on the lateral rectus muscle, while the optic nerve was spared, and no evidence of sinusitis. This report raised questions regarding the nature of the disease (Fig. 2). It was also prudent to rule out other soft tissue mass lesions such as an infected dermoid cyst which is a common entity with respect to the site of swelling or any enlarging vascular lesion, for example. Hence, the patient underwent a magnetic resonance imaging (MRI) of the nose, paranasal sinuses (PNS) and orbits. 

The case history along with the radiological and clinical findings confirmed the presence of a well-circumscribed thick-walled abscess in the right orbit impinging on the right lateral rectus muscle (Fig.2). 

**Fig 3:**
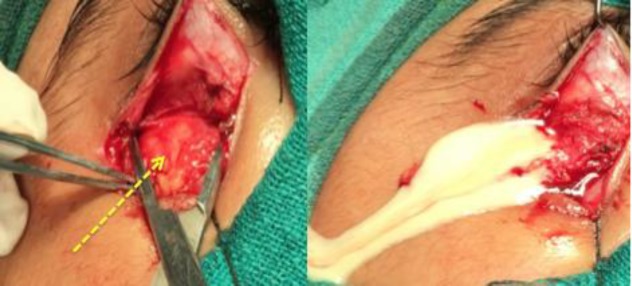
Intraoperative photograph showing the well defined cystic cavity (arrow) and free flow of pus after incision over the cavity

Following this conclusion, the patient was subjected to orbital abscess drainage under general anesthesia. An incision was given 5 mm below the right lower eyelid and the soft tissue dissected. A thick-walled cystic swelling was encountered after dissecting through the orbital fat. On aspiration, the swelling expressed pus. About 6 ml of pus was evacuated after incising the abscess cavity wall (Fig.3). 

**Fig 4:**
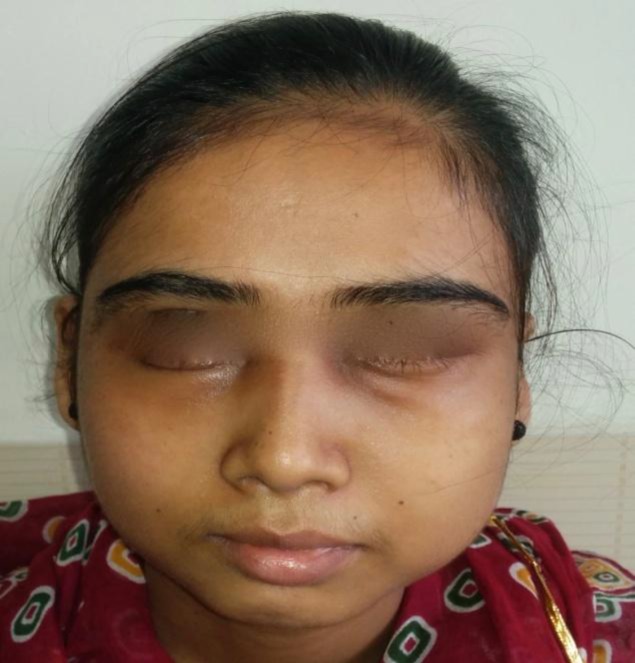
Postoperative photograph of the patient on the 7^th^ day

The cavity was flushed with saline and povidone iodine and the incision site was closed in layers. Pus was sent for microbiological analysis and culture but revealed no growth after 48 hours of culture. The patient was continued on broad-spectrum intravenous antibiotics for 7 days. The post-operative period was uneventful with slight chemosis, which settled within 48 hours. The patient maintained normal vision as before with obvious diminution in the size of the swelling (Fig.4). The patient was discharged on the 7^th^ postoperative day after suture removal.

## Discussion

Orbital abscesses occur secondary to direct contamination of eye; i.e. in cases of trauma to the eye or infected penetrating cornea surgeries. The infection to the orbit may also occur secondary to dental or sinonasal infections, surgical manipulation, or varicella infection, for example. However, orbital abscess in most patients presents as a complication of acute sinusitis (1). The orbital abscess that was confronted in this case was due to an odontogenic cause. This entity poses an imminent threat to the vision of the patient, mandating immediate management of the condition. The presentation may range from periorbital swelling, proptosis, restriction of ocular mobility, and pain to permanent impairment of visual acuity (2). If worsened, it may even progress to cavernous sinus thrombosis (3). Multiple pathways of the spread of an odontogenic infection to the orbit have been described. The maxillary sinus provides an easy route for the spread of the infection to the orbit via the inferior orbital fissure or any preformed breach in the orbital floor (4,5). The infection may also spread to the periorbital tissues directly via the canine fossa or fascial planes over the thin buccal cortical plane that can easily be eroded (6). The infection may invade infratemporal fossa or pterygopalatine fossa and reach the orbit through the infraorbital fissure (5-7). Another pathway is the spread of infection through the facial thrombophlebitis, which includes retrograde spread from the ophthalmic veins. The inferior and superior ophthalmic veins anastomose with angular and facial veins at the medial canthus. The inferior ophthalmic veins pass through the inferior orbital fissure and communicate with pterygoid plexus of veins. These veins are valveless and pose no resistance to the spread of infection (5,6). The orbit is a closed cavity and is very well connected through a system of valveless veins to the face, nose and PNS (8). The anatomy potentiates rapid spread of the infection from these areas to the orbit.

The most common cultures from a typical odontogenic infection are mixed cultures of aerobes and anaerobes, with a majority of anaerobic bacteria. 

However, the most common microbial flora isolated from the blood or pus specimen of an orbital abscess are streptococcal species such as Streptococcus viridans, Streptococcus pneumonia, Streptococcusmilleri, Streptococcus pyogenes, and also Staphylococcus aureus and Hemophillus influenza type B (6,8,9). Conversely, in this case report, the cultures were sterile, probably because of the use of antibiotics prior to obtaining the pus specimen. 

In our patient, the periapical infection that could have been present previously or may have occurred due to the aseptic and unsterile practices of the quack appears to have spread by retrograde thrombophlebitis. 

If suspected, the complication of an orbital infection must be managed aggressively with broad-spectrum antibiotics. Complete orbital and ophthalmological examination should be carried out along with relevant CT or MRI for the precise identification of the site, extent, and spread of the infection and presence of an abscess cavity. An abscess, if present, needs prompt drainage via an external approach. The drainage needs to be carried out very carefully due to the delicate contents of the orbit. 

In an Indian scenario, where illiteracy and poverty prevail, our case report shows how medical fraudsters are making a fortune out of naive people. The unsterile conditions in which they practice can lead to fatal complications such as cavernous sinus thrombosis from a simple procedure that needs to be carried out by a medical professional. Our patient was fortunate enough to report to us before any deterioration of vision, which could have happened if the abscess had led to pressure damage on the optic nerve or central retinal artery occlusion (CRAO). This illustrates the importance of prompt surgical drainage of the abscess to prevent an extremely poor outcome. The high cost associated with dental treatment, illiteracy, and a lack of adequate health professionals are the major reasons that compel people to consult quacks, leading to such misdiagnosis and mismanagement.

## Conclusion

Orbital complications secondary to an odontogenic cause are not common. This condition, if untreated in due time, carries the impending risk of loss of vision, rendering a vital organ non-functional. The patient may not present with a dental complaint in an acute presentation. Hence, the clinician must have a low threshold for suspicion for such an entity, which requires a multidisciplinary approach for aggressive management. Awareness needs to be raised among the patients regarding the quack culture prevalent in developing countries to prevent such unethical practices and life-threatening complications. 
